# Supplementary material for: Targets and genomic constraints of ectopic Dnmt3b expression
Source: eLife. 2018 Nov 23;7:e40757. doi: 10.7554/eLife.40757 (PMC6251628; doi:10.7554/eLife.40757)
Supplement: Supplementary file 1. [file elife-40757-supp1.docx]

Supplementary File 1

| **Primers for RT-qPCR** | | |
| --- | --- | --- |
| ***Dnmt3a*** | Forward_1 | 5'-CCTACTACATCAGCAAACGGAAAC-3' |
|  | Reverse_1 | 5'-GTTCTCTTCCACAGCATTCATTACT-3' |
|  | Forward_2 | 5'-GTTTTGACCTCCATAGAAGACACC-3' |
|  | Reverse_2 | 5'-CTTGGGGGTGTCACTGCTTT-3' |
| ***Dnmt3b*** | Forward_1 | 5'-ACCAGTGGTTAATAAGTCGAAGGT-3' |
|  | Reverse_1 | 5'-GACTCAGAAGCAGCAGAGTCATT-3' |
|  | Forward_2 | 5'-GTTTTGACCTCCATAGAAGACACC-3' |
|  | Reverse_2 | 5'-ACTTCATCATCTCTGTCTCCATCTC-3' |
|  | Forward_3 | 5'-GTGAATGACAAGAAAGACATCTCAA-3' |
|  | Reverse_3 | 5'-AGCTTATCATTCTTTGAAGCCATC-3' |
| ***Eed*** | Forward | 5'-CAGCCATTGTTTGGAGTTCA-3' |
|  | Reverse | 5'-TAACCGTATCTCCCCCTGTG-3' |
| **Primers for PCR with bisulfite converted DNA** | | |
| ***Uncx*** | Forward | 5'-'TGATGTTGATAAAGTAAAGY(C/T)G-3' |
|  | Reverse | 5'-CTCCAACCTACCTACAAACTTAAA-3' |
| ***Gal*** | Forward | 5'-GGTGAAAGGAATAGAGTTGTGA-3' |
|  | Reverse | 5'-CTAAAACTATAAATAATCCTCTC-3' |
| ***Gdf7*** | Forward | 5'-AGTATTTTGAGTTGTTTGGTT-3' |
|  | Reverse | 5'-CACACTTCCCCCATTATTAA-3' |
| ***Vsx2*** | Forward | 5'-TTTTTTTAAAGAATY(C/T)GGGAGATGA-3' |
|  | Reverse | 5'-CTCTACCTAATAACTACAAATAAAC-3' |
| **Primers for Dnmt3b mutution and isoform generation** | | |
| ***Dnmt3b* (C657G)** | Forward | 5'-TAGAGAGATCATTGCCTGGGCTTCCACCAATCACC-3' |
|  | Reverse | 5'-GGTGATTGGTGGAAGCCCAGGCAATGATCTCTCTA-3' |
| ***Dnmt3b* (R829G)** | Forward | 5'-ACGGGCGCCGCCGCCCATGTTGG-3' |
|  | Reverse | 5'-CCAACATGGGCGGCGGCGCCCGT-3' |
| ***Dnmt3b* (S277P)** | Forward | 5'-GTCAGCAGAGATCTCAGGAAACTTGCCATCACCAAA-3' |
|  | Reverse | 5'-TTTGGTGATGGCAAGTTTCCTGAGATCTCTGCTGAC-3' |
| ***Dnmt3b* (VWRR)** | Forward | 5'-GAAGCCCTTGATCTTTCCCCTCCTGAGGTCACCTATTCCAAACT-3' |
|  | Reverse | 5'-AGTTTGGAATAGGTGACCTCAGGAGGGGAAAGATCAAGGGCTTC-3' |
| ***Dnmt3b3*** | Forward_1 | 5'-GAGGGCCTCAAACCCAACAAGAAGCAACCAGAGAACAAAAGTCGAAGACGCACAACCAAT-3' |
|  | Reverse_1 | 5'-ATTGGTTGTGCGTCTTCGACTTTTGTTCTCTGGTTGCTTCTTGTTGGGTTTGAGGCCCTC-3' |
|  | Forward_2 | 5'-TTCTGGGGTAACCTACCCGGAATGAACAGGATCTTCGGCTTCCCTGCTCACTACACGGAC-3' |
|  | Reverse_2 | 5'-GTCCGTGTAGTGAGCAGGGAAGCCGAAGATCCTGTTCATTCCGGGTAGGTTACCCCAGAA-3' |
